# *TWIST1*/miR-584/*TUSC2* pathway induces resistance to apoptosis in thyroid cancer cells

**DOI:** 10.18632/oncotarget.12129

**Published:** 2016-09-20

**Authors:** Francesca Maria Orlandella, Gennaro Di Maro, Clara Ugolini, Fulvio Basolo, Giuliana Salvatore

**Affiliations:** ^1^ IRCCS SDN Spa, 80121 Napoli, Italy; ^2^ Dipartimento di Area Medica, Azienda Ospedaliero-Universitaria Pisana, 56126 Pisa, Italy; ^3^ Dipartimento di Patologia Chirugica, Medica, Molecolare e dell'Area Critica dell' Università di Pisa, 56126 Pisa, Italy; ^4^ Dipartimento di Scienze Motorie e del Benessere, Universita' “Parthenope”, 80133 Napoli, Italy

**Keywords:** miR-584, TWIST1, TUSC2, anaplastic thyroid carcinoma, apoptosis

## Abstract

*TWIST1*, a transcription factor, plays a pivotal role in cancer initiation and progression. Anaplastic thyroid carcinoma (ATC) is one of the deadliest human malignancies; *TWIST1* is overexpressed in ATC and increases thyroid cancer cell survival, migration and invasion. The molecular mechanisms underlying the effects of *TWIST1* are partially known. Here, using miRNome profiling of papillary thyroid cancer cells (TPC-1) ectopically expressing *TWIST1*, we identified miR-584. We showed that *TWIST1* directly binds miR-584 using chromatin immunoprecipitation. Importantly, miR-584 was up-regulated in human ATC compared to papillary thyroid carcinoma (PTC) and normal thyroid samples. Overexpression of miR-584 in TPC cells induced resistance to apoptosis, whereas stable transfection of anti-miR-584 in TPC-TWIST1 and 8505C cells increased the sensitivity to apoptosis. Using bioinformatics programs, we identified *TUSC2* (tumor suppressor candidate 2) as a novel target of miR-584. *TUSC2* mRNA and protein levels were decreased in TPC miR-584 and increased in TPC-TWIST1 anti-miR-584 cells. Luciferase assays demonstrated direct targeting. Restored expression of *TUSC2* rescued the inhibition of apoptosis induced by miR-584. Finally, qRT-PCR and immunohistochemical analysis showed that TUSC2 was down-regulated in ATC and PTC samples compared to normal thyroids. In conclusion, our study identified a novel *TWIST1*/miR-584/*TUSC2* pathway that plays a role in resistance to apoptosis of thyroid cancer cells.

## INTRODUCTION

*TWIST1* is a basic helix-loop-helix transcription factor essential for the development of mesoderm-derived tissues [1]. *TWIST1* reactivation has been observed in many human cancers, where it was correlated with poor prognosis [2, 3]. *TWIST1* promotes cancer progression [2] by inducing epithelial to mesenchymal transition (EMT) [4] and invadopodia formation [5]. *TWIST1* promotes a cancer stem cell phenotype [6, 7], inhibits apoptosis [8] and increases resistance to chemotherapy [2].

Thyroid carcinoma derived from follicular cells includes different malignancies, ranging from well-differentiated to undifferentiated (or anaplastic) carcinoma [9, 10]. The molecular mechanisms driving thyroid neoplastic progression are not fully understood [11]. Anaplastic thyroid carcinoma (ATC) is among the most lethal human cancers [12–17]. ATC cells show an infiltration of approximately 50% tumor-associated macrophages, which increase tumor aggressiveness [18, 19]. EMT and stemness play an important role in the pathogenesis of ATC [20–22]. *TWIST1* is up-regulated in ATC and increases cell migration, invasion and resistance to apoptosis [21, 23, 24].

Recently, we identified a set of mRNAs that mediates the biological effects of *TWIST1* in thyroid cancer cells [25]. *TWIST1* effects are also mediated by miRNAs [26–29]. miRNAs are a class of small nuclear RNA molecules that inhibit post-transcriptional gene expression. miRNAs have a central role in the establishment and progression of human cancers [30].

Here, using a miRNome screening analysis of papillary thyroid cancer cells (TPC-1, hereafter named TPC) ectopically expressing *TWIST1* compared to control cells, we found that miR-584 was up-regulated by *TWIST1*, and we studied its role in thyroid cancer cells.

## RESULTS

### *TWIST1* alters the expression of a set of miRNAs in papillary thyroid cancer cells

To identify miRNA targets of *TWIST1*, we carried out miRNome profiling of TPC cells transfected with *TWIST1* compared to vector control cells. We found 45 miRNAs that were differentially expressed (14 up-regulated and 31 down-regulated, Supplementary Tables S1 and S2) by more than 2-fold in TPC-TWIST1 cells.

**Figure 1:**
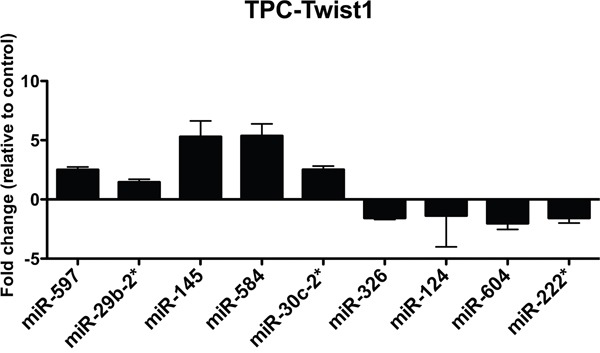
Expression levels of the indicated miRNAs in TPC-TWIST1 cells qRT-PCR analysis of the indicated miRNAs in TPC-TWIST1 cells compared to vector control cells. The expression level of each miRNA was normalized to the level of U6 snRNA, and fold changes were evaluated using the 2^− Δ Ct^ method, assuming that the value of the vector control cells is equal to 1. The average results of three independent experiments are reported with error bars indicating standard deviation.

To verify the microarray data, we performed qRT-PCR of the top 5 up-regulated and the top 5 down-regulated miRNAs in TPC-TWIST1 cells. We confirmed 90% of the deregulated miRNAs (Figure 1); only miR-190 was not changed (data not shown). Among the most up-regulated miRNAs, we focused on miR-584.

### miR-584 is transcriptionally regulated by *TWIST1*

**Figure 2:**
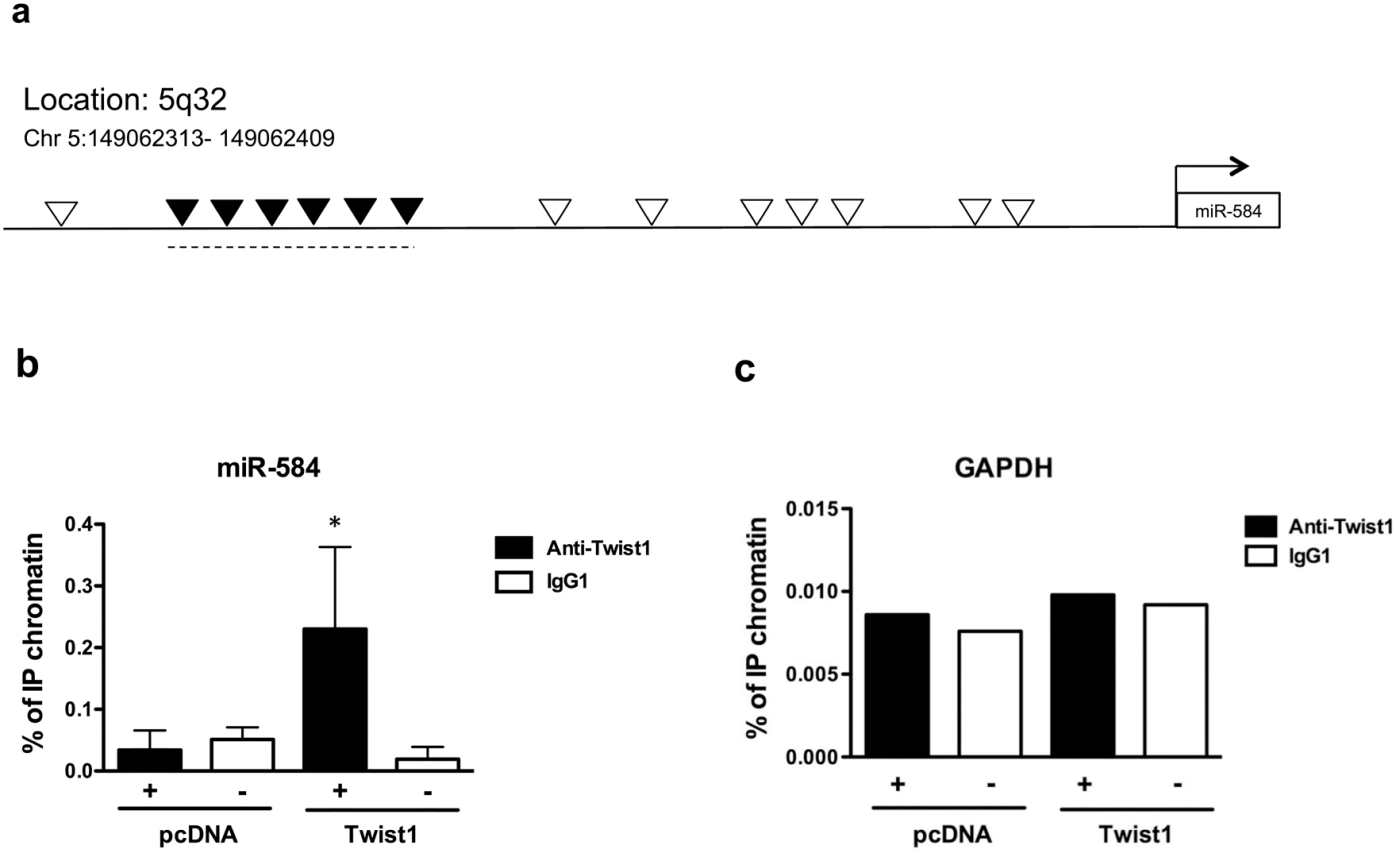
*TWIST1* directly binds a region upstream of miR-584 **a.** Schematic representation of all E-box binding sites (indicated as —) in the genomic locus of miR-584. Black ▼ indicate the E-boxes amplified in ChIP assays. **b.** ChIP assay, followed by qRT-PCR, was performed with TPC-TWIST1 or control (pcDNA) cells. Columns represent the average of four independent experiments □± SD. * *p*< 0.05. **c.** The amplicon of the GAPDH promoter was used as a negative control. The figure represents four independent experiments.

To determine whether *TWIST1* directly binds miR-584, we performed chromatin immunoprecipitation (ChIP). *TWIST1* binds to the E-box sequence motif (CANNTG) in its target genes [1]; thus we examined the 4-kb region upstream of miR-584 on chromosome 5q.32 and found 14 E-boxes (Figure 2a). Chromatin samples of TPC-TWIST1 or control cells (pcDNA) were cross-linked and immunoprecipitated with anti-TWIST1 or with control (IgG1) antibodies. A region containing six consecutives E-boxes was amplified by qRT-PCR in TPC-TWIST1 cells but not in control cells (Figure 2b). No amplification was obtained with anti-IgG1 precipitates (Figure 2b) or when primers for the control GAPDH promoter were used (Figure 2c).

### miR-584 is up-regulated in human ATC samples

**Figure 3:**
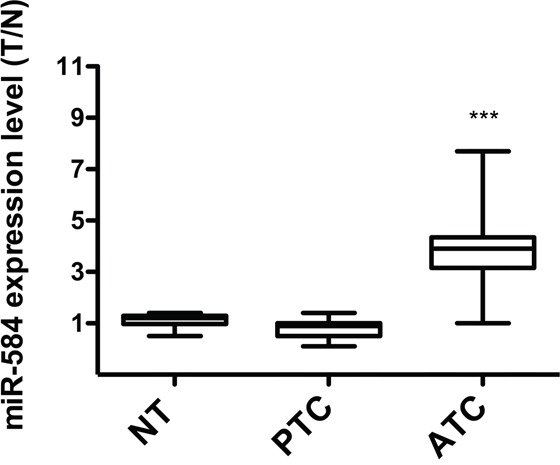
Expression of miR-584 in thyroid tissue samples qRT-PCR analysis of miR-584 in normal thyroid (NT) (n=10), PTC (n=11), and ATC (n=6) snap-frozen tissue samples. The expression level of miR-584 was normalized to the U6 snRNA levels. The level of miR-584 in each sample was measured by comparing its fluorescence threshold with the average fluorescence threshold of the NT samples. The average results of quadruplicate experiments are plotted. ***, *p*< 0.001.

To study the *in vivo* expression of miR-584, we measured miR-584 levels using qRT-PCR in 10 normal thyroid (NT), 11 PTC, and 6 ATC tissues samples. As shown in Figure 3, most of the ATC (~80%) samples overexpressed (~3-fold) miR-584 compared to the NT and PTC samples.

### Forced expression of miR-584 does not impair migration, invasion and proliferation of TPC cells

*TWIST1* sustains the invasive and migratory phenotype of thyroid cancer cells [21, 23]; therefore, we investigated whether miR-584 was in part responsible for these effects. Thus, we stably transfected TPC cells, in which the basal level of miR-584 is shown in Figure S1a, with a precursor of miR-584 or with an empty vector (miR-null). qRT-PCR confirmed that miR-584 was increased in a selected mass population (Figure S1b).

Next, we performed wound-healing assays in TPC miR-584 or TPC miR-null cells and monitored cell migration after 12 and 24 hours. As shown in Figure S2a, miR-584 did not influence the motility of TPC cells as both cell lines showed equal wound healing. We also performed a Matrigel invasion assay in which TPC miR-584 and control cells were seeded into the top chamber of transwells, and their ability to invade into the Matrigel was evaluated after 24 hours. As shown in Figures S2b and S2c, the TPC miR-584 and control cells had similar cell invasion abilities. Finally, we assessed whether miR-584 influenced cell proliferation rate. As shown in Figure S1d, both cell lines grew at a similar rate.

### miR-584 protects TPC cells from apoptosis induced by doxorubicin and staurosporine

**Figure 4:**
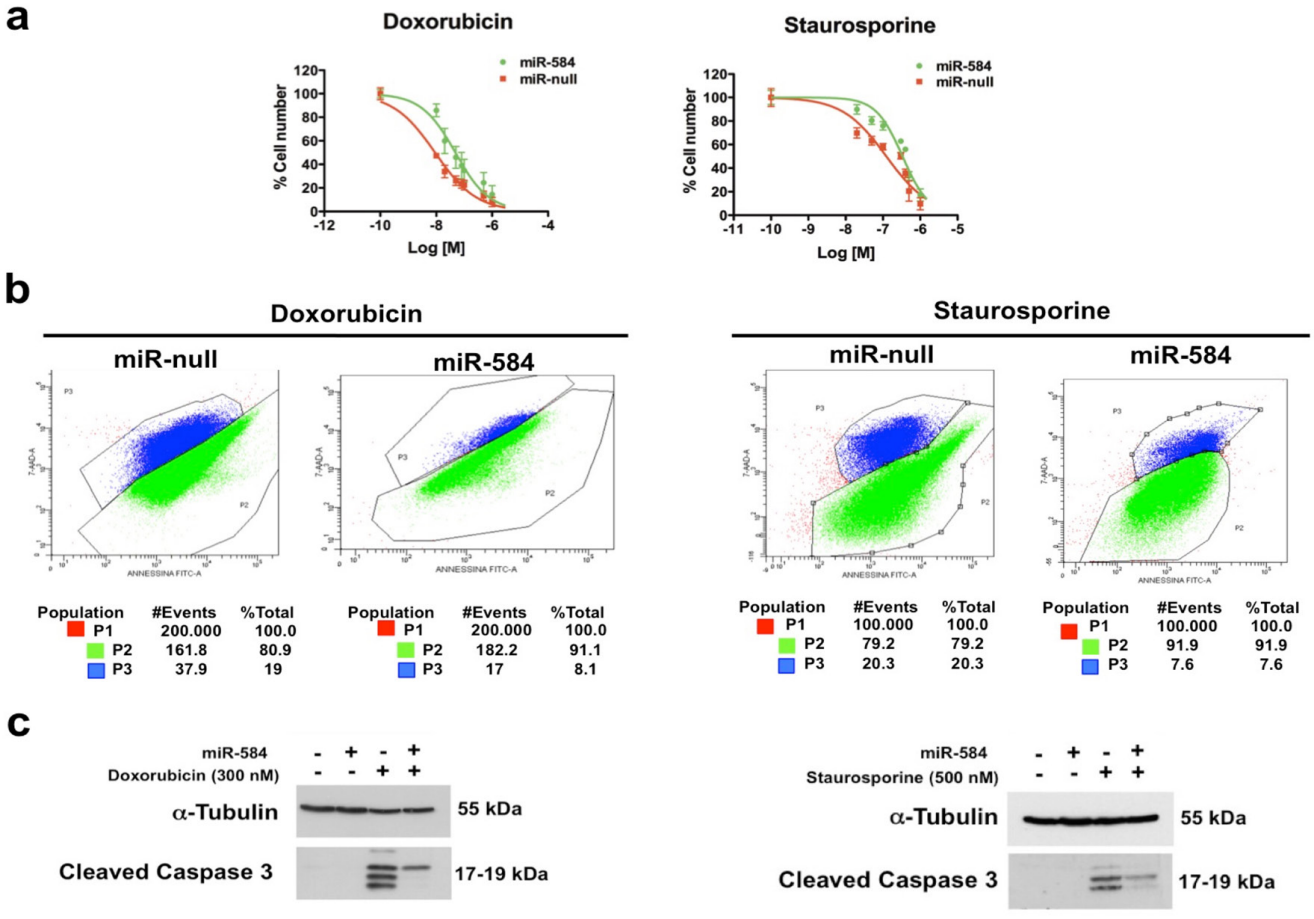
Effects of ectopic expression of miR-584 in TPC cells **a.** TPC miR-584 and TPC miR-null cell lines weretreated with different doses of doxorubicin and staurosporine for 48 and 24 hours, respectively, and counted in triplicate. The IC_50_ values were calculated with GraphPad software. The average of three independent experiments ± SD is shown. **b.** Dual staining of apoptotic cells with 7-AAD and Annexin V after treatment with doxorubicin and staurosporine. The percentages of apoptotic cells (P3) or of viable cells (P2) are indicated. **c.** TPC miR-584 and TPC miR-null cells were treated with 300 nM of doxorubicin or with 500 nM of staurosporine, lysed after 48 and 24 hours, respectively, and blotted with the antibodies for Cleaved CASPASE 3 or α-TUBULIN.

We investigated whether miR-584 affects the sensitivity to apoptosis because *TWIST1* is an anti-apoptotic factor [1, 2]. TPC miR-584 and control cells were treated with different doses of doxorubicin or staurosporine and counted after 48 and 24 hours, respectively. The 50% growth inhibitory concentration (IC_50_) of doxorubicin in TPC miR-null cells was 8.9 nM, while in TPC miR-584 cells, it was 48 nM (Figure 4a, left). Similarly, the IC_50_ of staurosporine in TPC miR-null cells was 123 nM, while in TPC miR-584 cells, it was 334 nM (Figure 4a, right).

To further confirm these findings, we treated TPC miR-584 and TPC miR-null cells with doxorubicin and staurosporine at IC_50_ values, stained them with 7AAD/Annexin V and analyzed the cells by flow cytometry. As shown in Figure 4b, the percentage of apoptotic cells (P3) in TPC miR-584 cells treated with doxorubicin was 8.1%, while in TPC miR-null cells, it was 19%. The percentage of apoptotic cells (P3) in TPC miR-584 cells treated with staurosporine was 7.6%, while in TPC miR-null cells, it was 20.3%.

Accordingly, TPC miR-584 cells showed decreased activation of cleaved CASPASE 3 compared to control cells following the treatments (Figure 4c). Overall, these results demonstrate that miR-584 protects TPC cells from apoptosis.

### miR-584 silencing in TPC-TWIST1 and 8505C cells increases sensitivity to apoptosis induced by doxorubicin and staurosporine

**Figure 5:**
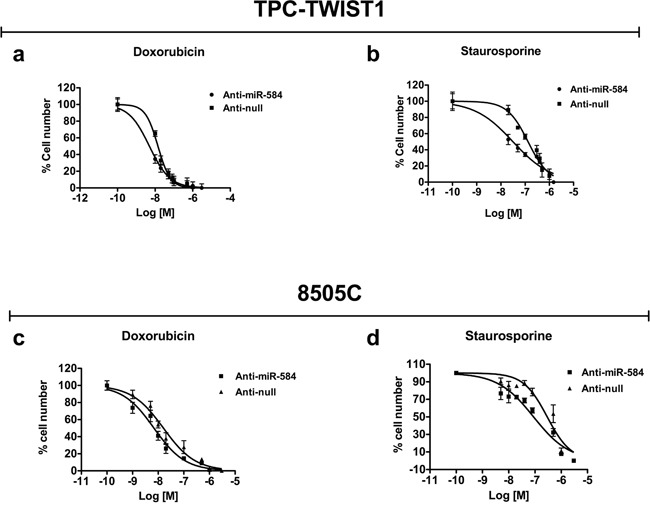
Effects of silencing of miR-584 in TPC-TWIST1 and 8505C cells The indicatedcells were treated with increasing doses of doxorubicin or staurosporine and counted after 48 and 24 hours, respectively. Values represent the average of four independent experiments ± SD.

To confirm these data, we performed the mirror experiment; thus, we stably silenced TPC-TWIST1 cells with anti-miR-584 or a control (anti-null). The silencing efficiency is shown in Figure S3a. miR-584 silencing in TPC-TWIST1 cells did not impair cell proliferation (Figure S3b), migration (Figure S3c) and invasion (Figures S3d and S3e). Then, we treated TPC-TWIST1 anti-miR-584 and anti-null cells with different doses of doxorubicin and staurosporine. As shown in Figure 5a and 5b, silencing of miR-584 in TPC-TWIST1 cells increased sensitivity to apoptosis. Then, we assessed whether silencing of miR-584 increases sensitivity to apoptosis in an ATC cell line characterized by a more aggressive phenotype. For this aim, we stably transfected 8505C cells harboring a BRAF V600E mutation, which expressed high endogenous levels of miR-584 (Figure S4a), with anti-miR-584 plasmid or control (anti- null). The silencing efficiency is shown in Figure S4b. Silencing of miR-584 in 8505C cells did not significantly affect cell migration, invasion and proliferation (Figure S5) but increased sensitivity to apoptosis following the treatments (Figure 5c and 5d), confirming the results obtained in TPC-TWIST1 cells.

### *TUSC2* is a direct target of miR-584

**Figure 6:**
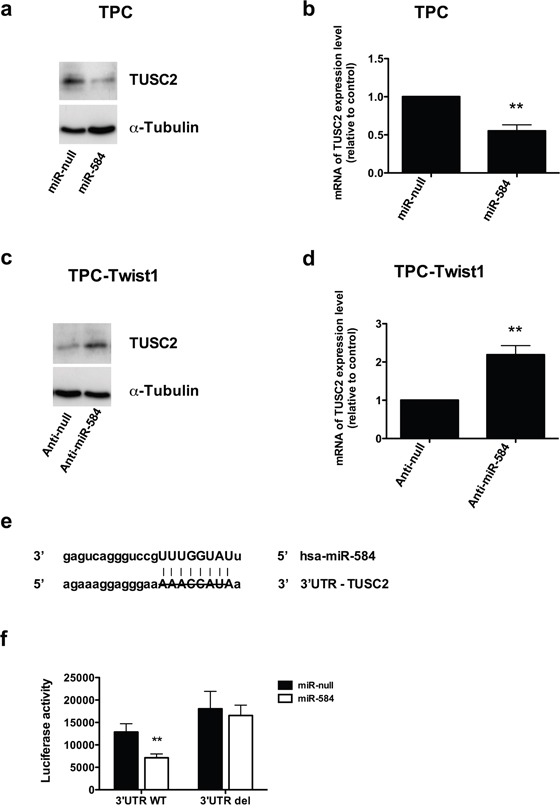
TUSC2 is a direct target of miR-584 **a.** Immunoblotting of TUSC2 and α-TUBULIN in TPC miR-584 or control cells. **b.** qRT-PCR of *TUSC2* in TPC miR-584 or control cells; data were normalized to the level of *POLR2A* assuming that the value of TPC miR-null is equal to 1. **c.** Immunoblotting of TUSC2 and α-TUBULIN in TPC-TWIST1 anti-miR-584 or control cells. **d.** qRT-PCR of *TUSC2* in TPC-TWIST1 anti-miR-584 or control cells. **e.** Predicted duplex formation between human 3′UTR-TUSC2 and miR-584;the strikethrough sequence of 3′UTR of TUSC2 corresponds to the predicted binding site of miR-584 deleted in the mutant 3′UTR-TUSC2 plasmid (3′UTR del). **f.** Luciferase activity of TUSC2 wild-type (3′UTR WT) or deleted (3′UTR del) reporter genes in TPC cells stably transfected with miR-584 or control. Luciferase activity was normalized with a co-transfected β-actin plasmid. **, *p*< 0.01.

miRNAs are negative regulators of gene expression. Using the bioinformatics algorithm TargetScan, we selected *TUSC2* (tumor suppressor candidate 2) among the top predicted targets of miR-584 for further analysis. This prediction was also confirmed using other bioinformatics programs (PicTar, DIANA Lab, PITA, RNA22, RNAhybrid, miRDB and miRanda). *TUSC2* is down-regulated in several human cancers and induces apoptosis [31]. Initially, we evaluated TUSC2 expression in TPC miR-584 and in TPC-TWIST1 anti-miR-584 cells and controls. As shown in Figure 6a, TUSC2 protein levels were decreased in TPC miR-584 cells compared to TPC miR-null cells. *TUSC2* was also down-regulated at the mRNA level (Figure 6b), suggesting that miR-584 both reduced translation and accelerated mRNA degradation of *TUSC2*. Similarly, TUSC2 protein and mRNA levels were increased compared to the controls in TPC-TWIST1 anti-miR-584 cells (Figure 6c and 6d).

To confirm that miR-584 directly regulates *TUSC2*, we performed a luciferase reporter assay in which we transfected TPC miR-584 and TPC miR-null cells with a vector containing a luciferase reporter gene fused with the 3′ untranslated (UTR) region of *TUSC2*, which was driven by a constitutive promoter. We also transfected these cells with a mutant version of this plasmid, where the predicted miR-584 binding site of *TUSC2* was deleted (Figure 6e). As shown in Figure 6f, the luciferase activity was decreased after transfection of a 3′UTR vector containing *TUSC2* wild type (3′UTR WT). This was reversed when the putative miR-584 binding site in the TUSC2 3′-UTR (3′UTR del) was deleted (Figure 6f).

### *TUSC2* rescues the resistance to apoptosis induced by miR-584

**Figure 7:**
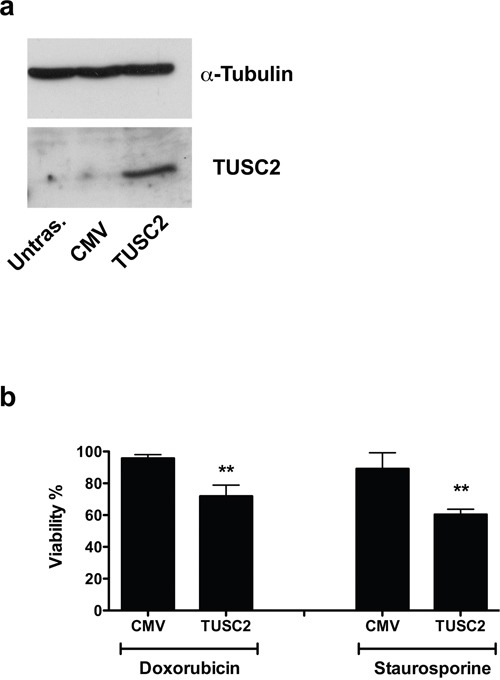
TUSC2 partially rescues the phenotype induced by miR-584 **a.** TPC miR-584 cells were transiently transfected with TUSC2 plasmid or with the vector control (CMV) and analyzed for TUSC2 protein expression by Western blotting. An antiα-TUBULIN antibody was used for normalization. **b.** TPC miR-584 cells were transfected with TUSC2 or vector controlandtreated with 50 nM of doxorubicin and 300 nM of staurosporine; after 48 and 24 hours, respectively, cells were stained with trypan blue and counted in triplicate. The percentage viability± SD is shown, **, *p* < 0.01.

To verify if *TUSC2* is involved in resistance to apoptosis induced by miR-584, we transiently transfected TPC miR-584 cells with a construct that expresses *TUSC2* but lacks the 3′UTR of the TUSC2-encoding mRNA (thus, the TUSC2 mRNA is refractory to inhibition of translation induced by miR-584) or with a control vector (CMV). Efficiency of transient transfection is shown in Figure 7a. As shown in Figure 7b, TPC miR-584 cells transfected with TUSC2 and treated with doxorubicin (50 nM) or staurosporine (300 nM) showed decreased cell viability compared with control cells (CMV). Thus, *TUSC2* partially rescued the phenotype induced by miR-584 in TPC cells. Overall, these results demonstrate that *TUSC2* is a functional target of miR-584.

### TUSC2 is down-regulated in thyroid carcinoma samples

**Figure 8:**
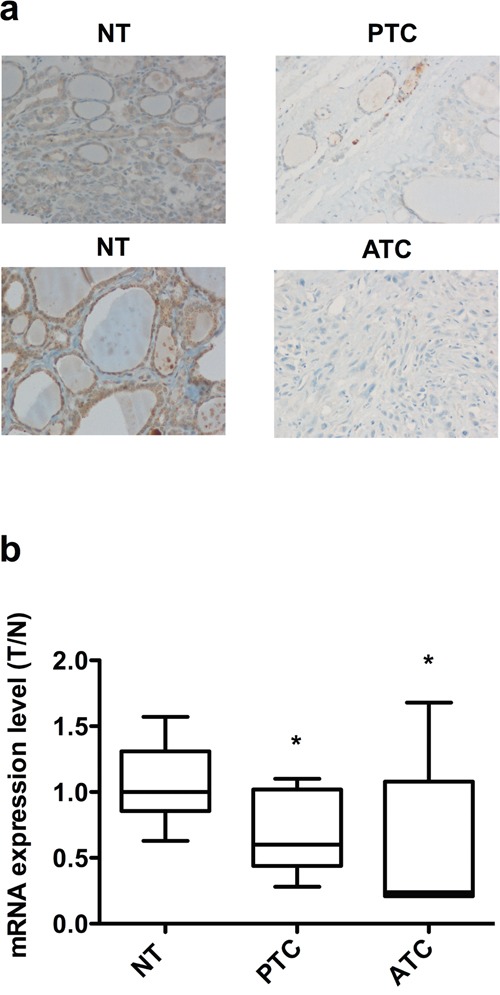
Expression of TUSC2 in thyroid tissue samples **a.** Immunohistochemical analysis of TUSC2 protein expression in normal and malignant thyroid tissues. Representative sections (20X magnification) of PTC, ATC and contralateral NT stained with an anti-TUSC2 antibody are shown. **b.** qRT-PCR of TUSC2 in NT (n=10), PTC (n=11) and ATC (n=6) snap-frozen tissue samples. The expression level of TUSC2 in each sample was measured by comparing its fluorescence threshold with the average fluorescence threshold of the NT samples. The average results of quadruplicate samples are plotted. The expression level in each sample was normalized with the β-Actin measurement. * *p*< 0.05.

Finally, we evaluated the expression level of TUSC2 in 37 normal thyroid (NT), 20 PTC and 40 ATC samples using immunohistochemistry. As shown in Table 1, all normal thyroids were positive for TUSC2, with ~60% of the samples showing strong TUSC2 staining. In contrast, TUSC2 was undetectable in almost all ATC samples. The majority of PTC samples were negative for TUSC2, suggesting a correlation between TUSC2 down-regulation and tumor progression. Representative images of TUSC2 staining in PTC, ATC and normal contralateral thyroid lobes are shown in Figure 8a. We also evaluated *TUSC2* mRNA levels by qRT-PCR in an independent set of samples including 10 NT, 11 PTC and 6 ATC. *TUSC2* mRNA was down-regulated in the majority of ATC and in a fraction of PTC samples (Figure 8b).

**Table 1:** TUSC2 protein expression in thyroid samples (n = 97)

TUSC2	NT (N = 37)	PTC (N = 20)	ATC (N = 40)
+++	22 (59.5%)	-	-
++	11 (29.8%)	1 (5%)	-
+	4 (10.8%)	4 (20%)	1 (2.5%)
Negative	-	15 (75%)	39 (97.5%)

+, ≥ 5 to ≤ 25% of positive cells; ++, > 25 to 60% of positive cells; +++ ≥ 60% of positive cells.

## DISCUSSION

miRNA targets of Twist1 have not been fully elucidated. Here, we identified a set of potential miRNAs targets of Twist1 in thyroid cancer cells.

Since deregulation of miRNAs has been described in thyroid carcinogenesis [19, 32, 33], we searched in our array of TPC-TWIST1 cells miRNAs previously reported to be deregulated in thyroid carcinoma samples. We found a decrease of miR-301a and miR-200c in TPC-TWIST1 cells (Table S2); these miRNAs were reported to be down-regulated in ATC [34].

Additionally, we found that miR-145 and miR-101 were up-regulated in TPC-TWIST1 cells; these miRNAs, in contrast, were down-regulated in ATC samples [34–38]. This difference could be explained by the fact that TPC-TWIST1 is a cell line and thus not completely representative of cancer complexity.

Consistent with our findings, miR-584 was also shown to be up-regulated in a microarray screening of different malignant thyroid tissues compared to benign thyroid lesions [39]. Furthermore, Xiang recently reported that transient forced expression of miR-584 in K1 cells had no effect on cell proliferation but decreased cell migration [40]. It is possible that the different results obtained for cell migration compared to our study is due to the different cell lines used and to different transfection methods.

Here, we found that miR-584 was up-regulated in ATC, while it is down-regulated in glioma, where it suppress cell growth [41]; in renal cell carcinoma, where it targets *ROCK1* to decrease invasion [42]; and in breast carcinoma [43]. The differential expression of miR-584 in several human cancers may be because the functions of miRNAs are dependent on cellular context, which is due to the different expression of their targets mRNAs. Accordingly, some miRNAs are oncogenic and up-regulated in one cancer type but are tumor suppressors and down-regulated in another cancer type [30].

Using different computational programs, we identified *TUSC2* as a novel target of miR-584. *TUSC2* is located on chromosome 3p21.3, a region in which deficient gene expression is observed in lung cancers [44, 45]. In human lung cancer cell lines, *TUSC2* induced apoptosis [46]. Overexpression of *TUSC2* inhibited tumor growth and progression in mouse models [47], while *TUSC2* knockout mice showed an increased frequency of spontaneous cancers [48, 49].

Here, we showed that *TUSC2* partially reverts the phenotype induced by miR-584; thus, it is likely that other targets mediate the effects of miR-584 in TPC cells. Indeed, it is well known that miRNAs act by inhibiting multiple targets.

Moreover, we demonstrated by immunohistochemistry analysis that TUSC2 was down-regulated not only in ATC but also in a percentage of PTC samples, which did not express miR-584. This suggests that other mechanisms could control the expression of TUSC2 in PTC samples where TUSC2 is down-regulated. Consistent with this hypothesis, recent data revealed several mechanisms of regulation of *TUSC2*: different miRNAs (named miR-98, -93 -197 and -378) have been identified as negative regulators of *TUSC2* [50, 51]; methylation of *TUSC2* was identified in head and neck tumors [52]; myristoylation of the TUSC2 protein has been reported in lung cancers [53]; and two *TUSC2* pseudogenes were identified as regulators of *TUSC2* activities [54].

Importantly, TUSC2-expressing nanoparticles have been tested in clinical trials in patients with lung cancer and showed anti-tumor activity and no significant side effects [55]. New therapeutic approaches for treating ATC are needed. Our data suggest that miR-584 and *TUSC2* could be used as therapeutic targets for ATC and as novel biomarkers of therapeutic response.

## MATERIALS AND METHODS

### Cell culture

The TPC-1 (named TPC) cell line was obtained from M. Nagao (Carcinogenesis Division, National Cancer Center Research Institute, Tokyo, Japan), and the 8505C cell line was purchased from DSMZ (Deutsche Sammlung von Mikroorganismen und Zellkulturen GmbH, Braunschweig, Germany). TPC and 8505C cells were grown in Dulbecco's modified Eagle's medium (DMEM) (Thermo Scientific, Waltham, MA, USA) containing 10% fetal bovine serum (FBS), L-glutamine and penicillin/streptomycin (Thermo Scientific). The non-transformed human thyroid follicular epithelial cell line Nthy-ori 3-1 was purchased from Sigma-Aldrich (St. Louis, MO, USA) and grown in RPMI-1640 medium supplemented with 10% FBS (Thermo Scientific). The cell lines were authenticated by short-tandem repeat profiling performing by BMR Genomics (http://www.bmr-genomics.it) in 2015. TPC-TWIST1 cells (mp1, mp2, and Cl2) were generated and characterized previously [23]. TPC-TWIST1 mp1 was used in all the experiments.

### Tissue samples

Tumors and normal thyroid tissue samples used for immunohistochemical analysis and quantitative RT-PCR were obtained from the Department of molecular, medical, surgical pathology and of critical areas, University of Pisa (Italy). Informed consent was obtained from each subject. The clinicopathological information for the PTC samples is listed in Table S3.

### Immunohistochemistry analysis

Immunohistochemical analysis of TUSC2 expression was performed on formalin-fixed, paraffin-embedded (FFPE) tumor sections of 60 tumor cases, including 40 ATC and 20 PTC samples. As a control, thyroid tissues from 8 normal glands of patients who underwent surgery for head and neck tumors and 29 normal contralateral tissues were stained. Rabbit polyclonal anti-human TUSC2 antibody (Proteintech Group, Inc. Chicago, USA) was used (dilution 1:150). Sections were stained using a Ventana automated slide stainer (Ventana Medical Systems, Tucson, Az). TUSC2 expression in epithelial tumor and normal tissue cells was evaluated independently by two investigators (C.U. and F.B.) who were blinded to the clinicopathological data. Discrepancies between the two observers were discussed with a third pathologist (A.P.). For all cases, the percentage of positive cells per 5 high power fields (40X) was determined.

### Microarray analysis

Total RNA was extracted from TPC-TWIST1 (TWIST1 mp1, TWIST1 mp2, TWIST1 Cl2) and pcDNA control cells. miRNA analysis was performed using TaqMan® LDA Cards (Thermo Scientific, Waltham, MA, USA) according to standard procedures and conducted at Aarhus Biotechnology (Aarhus, Denmark).

### RNA extraction and qRT-PCR

Total RNA was isolated using the mirVana™ miRNA Isolation Kit (Thermo Scientific, Waltham, MA, USA) according to the manufacturer's instructions. RNA samples were quantified using a NanoDrop spectrophotometer (Thermo Scientific) and verified by a 2100 Bioanalyzer (Agilent Technologies, Santa Clara, CA, USA); only samples with RNA integrity number (RIN) values > 7 were used for further analysis.

For miRNA detection, specific primers and probes (Thermo Scientific) were used. Briefly, 10 ng of total RNA was reverse transcribed using a miRNA Reverse Transcription Kit (Thermo Scientific), followed by amplification using a TaqMan Universal Master Mix II (Thermo Scientific). U6 snRNA was used as an endogenous control.

For gene expression analysis, the mRNA level of *TUSC2* was measured by quantitative PCR assays using specific primers and probes (Thermo Scientific) and TaqMan Universal PCR Master Mix (Thermo Scientific). *β-ACTIN* and *POLR2A* were used as endogenous controls for tumor samples and cell lines, respectively. PCR reactions were performed in triplicate, and fold changes were calculated with the formula: 2^−(sample 1 Δ Ct - sample 2 Δ Ct)^, where ΔCt is the difference between the amplification fluorescence thresholds of the gene of interest and the endogenous control used as an internal reference.

### Chromatin immunoprecipitation assay

Chromatin immunoprecipitation (ChiP) assays were performed according to the instructions of the ChiP Assay Kit (Merck, Billerica, MA, USA) and as previously described [25]. Briefly, TPC-pcDNA and TPC-TWIST1 cell lysates were sonicated on ice 30 times for 30 seconds each at the maximum settings. Samples were subjected to immunoprecipitation with anti-TWIST1 antibody (Twist2C1a, sc-81417; Santa Cruz Biotechnology, Dallas, USA) or control antibody (mouse IgG1 isotype control, clone G3A1; Cell Signaling Technology, Beverly, USA). For qRT-PCR, 2 of 30 μL immunoprecipitated DNA was used. Input DNA values were used to normalize the values from the quantitative ChIP samples. Percent input was calculated as 2^− Δ Ct x 3^, where Ct is cycle threshold and Δ Ct is the difference between Ct of input and Ct of immunoprecipitated samples. ChIP values represent the average of three independent experiments, and for each experiment qRT-PCR was performed in triplicate. The GAPDH promoter amplicon was used as a negative control. The sequences of the primers covering E-box regions used in ChIP assays were as follows:

miR-584 Forward Primer: 5′-tgcaatgtgtgtgttagcca-3′;

miR-584 Reverse Primer: 5′-atcattgctccttggctggt-3′;

GAPDH Forward Primer: 5′-cccaaagtcctcctgtttca-3′;

GAPDH Reverse Primer: 5′-gtcttgaggcctgagctac-3′.

### Cell transfections

The miR-584 precursor construct expressing pre-miR-584 (pEP-hsa-miR-584) and the corresponding empty vector (pEP-miR, named miR-null) were purchased from Cell Biolabs (San Diego, USA). For miRNA silencing, the hsa-miR-584 inhibitor and control vector (named anti-miR-584 and anti-miR-null, respectively) were purchased from GeneCopoeia (Labomics, Nivelles, Belgium).

To generate stable transfectants 1x10^5^ cells were incubated with 50 nmol/ml of plasmid (pEP-hsa-miR-584 or hsa-miR-584 inhibitor) or with their respective controls and transfected using Lipofectamine 2000 (Thermo Scientific) according to the manufacturer's instructions. After 48 hours, cell populations and clones were selected in 1 mg/ml puromycin (Sigma-Aldrich, St. Louis, MO, USA) and analyzed for miR-584 expression by qRT-PCR. One cell mass population was selected and used in all the experiments.

For transient transfection, a TUSC2 plasmid, which expresses TUSC2 but lacks the 3′UTR of TUSC2-encoding mRNA, and a scrambled control (named CMV) were purchased from GeneCopoeia (Nivelles, Belgium). TPC miR-584 cells were transiently transfected using Lipofectamine 2000 according to the manufacturer's instructions. TUSC2 expression was evaluated by Western blot analysis.

### Cell proliferation, migration and invasion assays

For cell proliferation rate, 5x10^4^ cells were plated in 6-well plates and counted in triplicate every 24 hours for 4 days.

Cell migration and invasion, which were assessed by wound-healing and Matrigel matrix (BD Biosciences, San Jose, CA, USA) assays, respectively, were evaluated as previously described [25].

### Apoptosis assays

Cells were plated in triplicate in 6-well plates at a density of 5x10^5^ cells and kept in DMEM supplemented with 10% FBS. The day after plating, the medium was replaced by fresh medium with or without various concentrations of doxorubicin (10 nM, 20 nM, 50 nM, 80 nM, 100 nM, 0.5 μM, 1 μM, 3 μM) (Sigma-Aldrich) and staurosporine (20 nM, 50 nM, 100 nM, 0.3 μM, 0.4 μM, 0.5 μM, 1 μM, 1.5 μM) (Sigma-Aldrich). To estimate the IC_50_ value, cells were counted with a TC10™ Automated Cell Counter (Bio-Rad, Richmond, VA, USA) after 24 or 48 hours of treatment with staurosporine and doxorubicin, respectively. IC_50_ values were calculated using GraphPad Prism software (La Jolla, CA, USA).

For determination of cell viability, cells were collected by trypsinization, stained for 10 min with 0.4% trypan-blue (Sigma-Aldrich) according to manufacturer's instructions, and counted in triplicate.

Cell death was determined by fluorescence-activated cell sorting (FACS) analysis using 7-amino-actinomycin (7-AAD) for dead cells and Annexin V (Beckman Coulter, Miami, FL, USA) for apoptotic cells according to the manufacturer's protocol. Viable cells stained negative for 7-AAD and Annexin V.

### Western blot

Immunoblotting was carried out according to standard procedures. Membranes were probed with the indicated antibodies: rabbit polyclonal anti-TUSC2 (Proteintech) diluted 1:200; rabbit polyclonal anti-Cleaved (Asp175) CASPASE 3 (Cell Signaling Technology) diluted 1:500; mouse monoclonal anti-α TUBULIN (Sigma-Aldrich) diluted 1:10000. Secondary anti-mouse and anti-rabbit antibodies coupled to horseradish peroxidase were purchased from Bio-Rad and diluted 1:3000. Immune complexes were developed by an enhanced chemiluminescence detection kit (Thermo Scientific).

### Luciferase reporter assay

TPC miR-584 cells were seeded into a 96-well plate (1x10^3^ cells per well) and kept in DMEM supplemented with 10% FBS. After 24 hours, the pLightSwitch 3′UTR reporter plasmids (pLightSwitch-TUSC2-3′UTR or pLightSwitch-Empty-3′UTR and pLightSwitch-TUSC2-3′UTR del) were transfected into the cells using FuGENE Transfection Reagent (Roche, Basel, Switzerland) according to the manufacturer's protocol. Luciferase activity was measured 24 hours after transfection using the LightSwitch Luciferase Assay reagent (SwitchGear Genomics, CA, USA) according to the manufacturer's protocol.

Deletion of the 3′UTR of TUSC2 was introduced in the pLightSwitch-TUSC2-3'UTR WT plasmid using the QuikChange site-directed mutagenesis kit (Agilent Technologies). The following oligonucleotides were used:

TUSC2-3′UTR del Forward: 5′- GGGACTGTTCACCACCTTGT- 3′;

TUSC2-3′UTR del Reverse: 5′- CCCAAGCCATTTCCCACATT-3′.

### Statistical analysis

Results were analyzed with unpaired t tests using GraphPad Prism 5 software (La Jolla, CA, USA) and are shown as the mean ± standard deviation (SD). Differences were considered significant when *p* < 0.05.

## SUPPLEMENTARY FIGURES AND TABLES


