# Supplementary material for: TWIST1/miR-584/TUSC2 pathway induces resistance to apoptosis in thyroid cancer cells
Source: Oncotarget. 2016 Sep 20;7(43):70575–88. doi: 10.18632/oncotarget.12129 (PMC5342575; doi:10.18632/oncotarget.12129)
Supplement: Supplementary file 1 [file oncotarget-07-70575-s001.pdf]

TWIST1/miR-584/*TUSC2* pathway induces resistance to apoptosis in thyroid cancer cells

SUPPLEMENTARY FIGURES AND TABLES

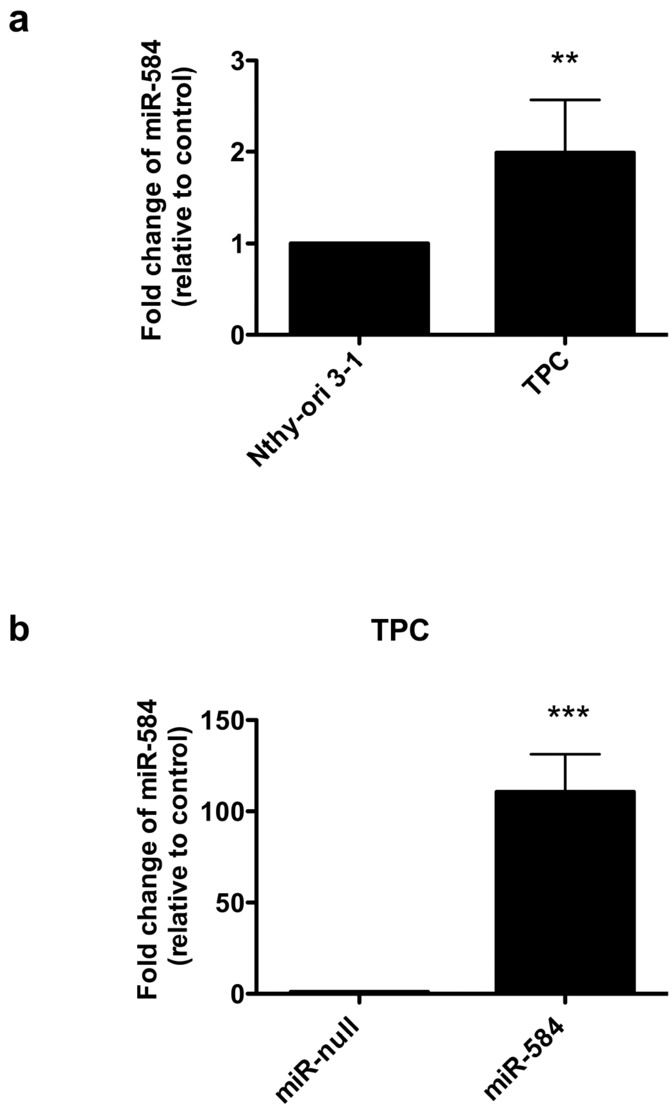

Supplementary Figure S1: Expression level of miR-584 in TPC cell line. a. The basal level of miR-584 in TPC cells was measured, by qRT-PCR, compared to non-transformed human thyroid cells (Nthy-ori 3-1). The expression levels of miR-584 were normalized to those of U6 snRNA. The average results of three independent experiments ± SD are plotted (**p <0.01). **b.** miR-584 expression level was measured by qRT-PCR in TPC cells after stable transfection with miR-584 plasmid; results are reported as fold change compared with the control (TPC miR-null). The expression levels of miR-584 were normalized to those of U6 snRNA. Values represent the average of three independent experiments ± SD (***p < 0.001).

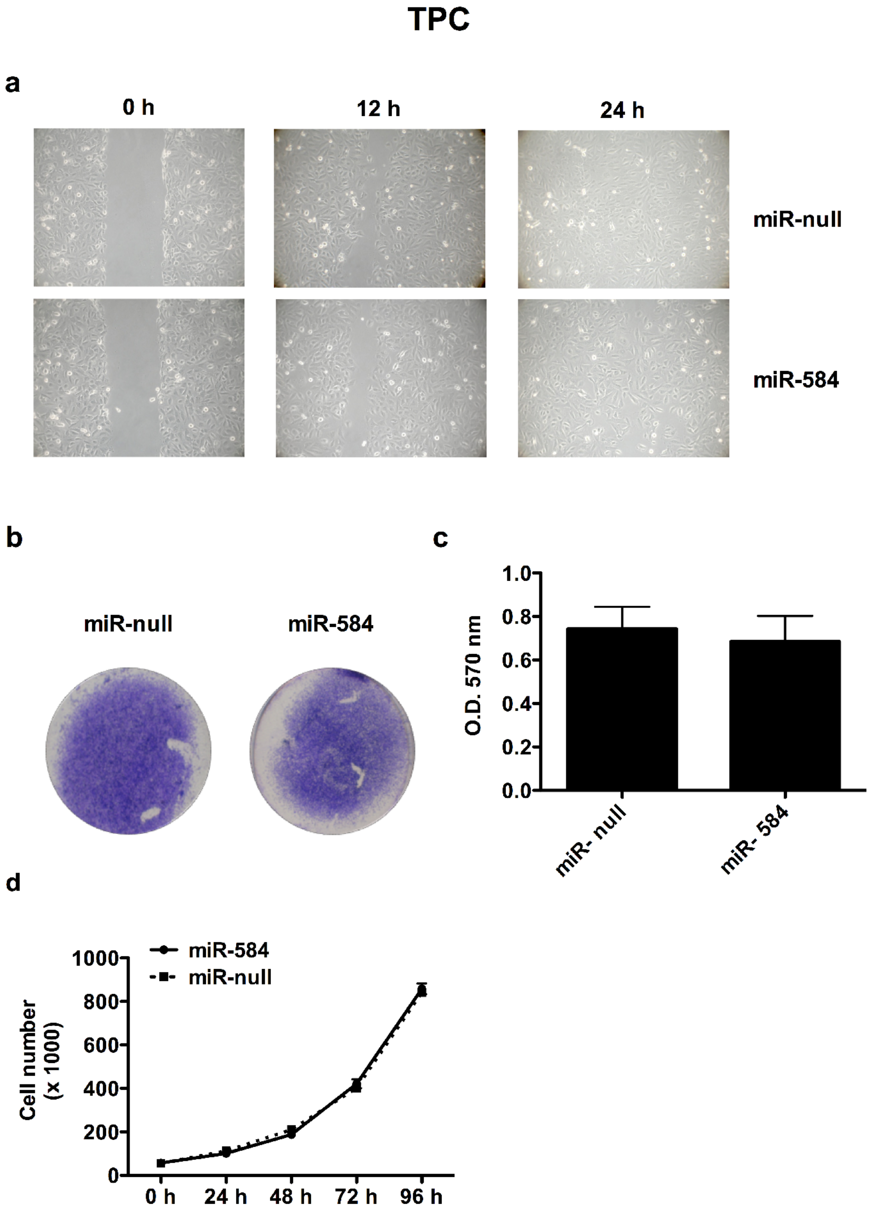

Supplementary Figure S2: Effects of miR-584 expression in TPC cells on cell migration, invasion and proliferation. **a.** Scratch wounds were inflicted at confluent cell monolayer of TPC miR-584 and of TPC miR-null; after 12 and 24 hours cells were photographed. **b-c.** TPC miR-584 and TPC miR-null were seeded in upper chamber of transwells, allowed to migrate for 24 hours, stained and photographed (b); the invasive ability into the Matrigel is expressed as absorbance at O.D. 570 nm (c). **d.** TPC miR-584 and TPC miR-null cells were plated and counted in triplicate every 24 hours for 4 days. Values represent the average of three independent experiments ± SD.

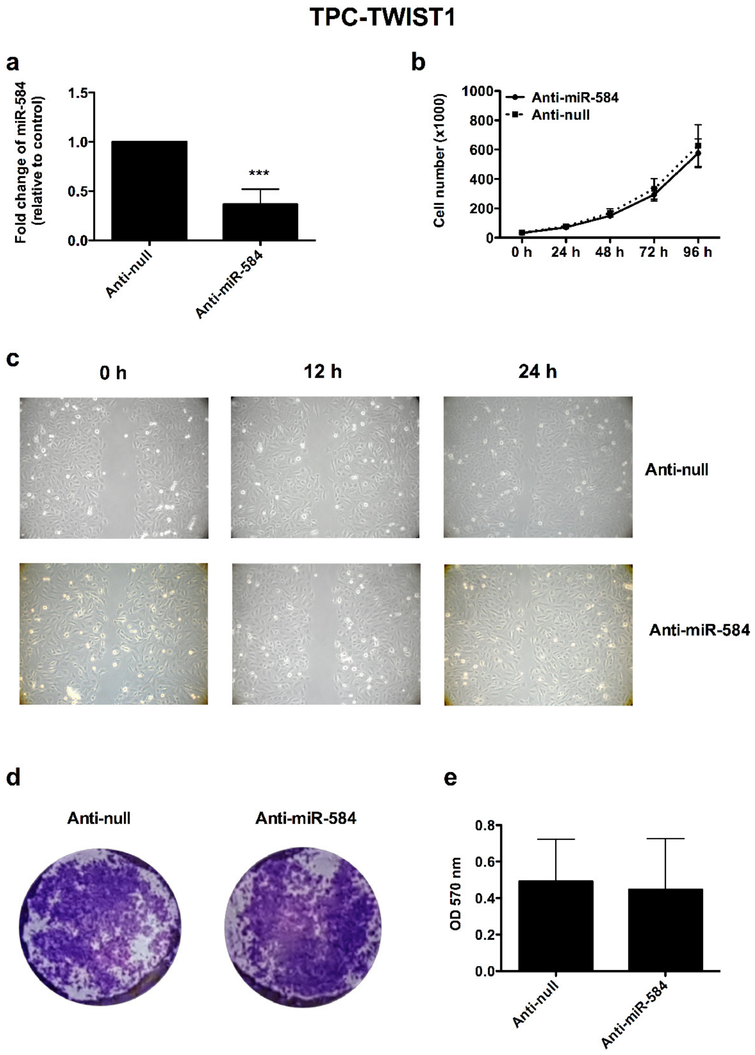

Supplementary Figure S3: Effects of miR-584 silencing in TPC-Twist1 cells on cell migration, invasion and proliferation. **a.** qRT-PCR of miR-584 in TPC-Twist1 cells transfected with anti-miR-584 or a empty vector (anti-null) are shown. The expression levels of miR-584 were normalized to those of U6 snRNA. Values represent the average of three independent experiments ± SD (***, $p < 0.001$). **b.** TPC-Twist1transfected cells were plated and counted at different time points. Values represent the average of three independent experiments ± SD. **c.** Scratch wounds were inflicted at confluent cell monolayer and after 12 and 24 hours cells were photographed. **d-e**. TPC-Twist1 anti-miR-584 and control cells were seeded in upper chamber of transwells, allowed to migrate for 24 hours, stained and photographed (d); invasion ability into the Matrigel is expressed as absorbance at O.D. 570 nm. Values represent the average of three independent experiments ± SD.

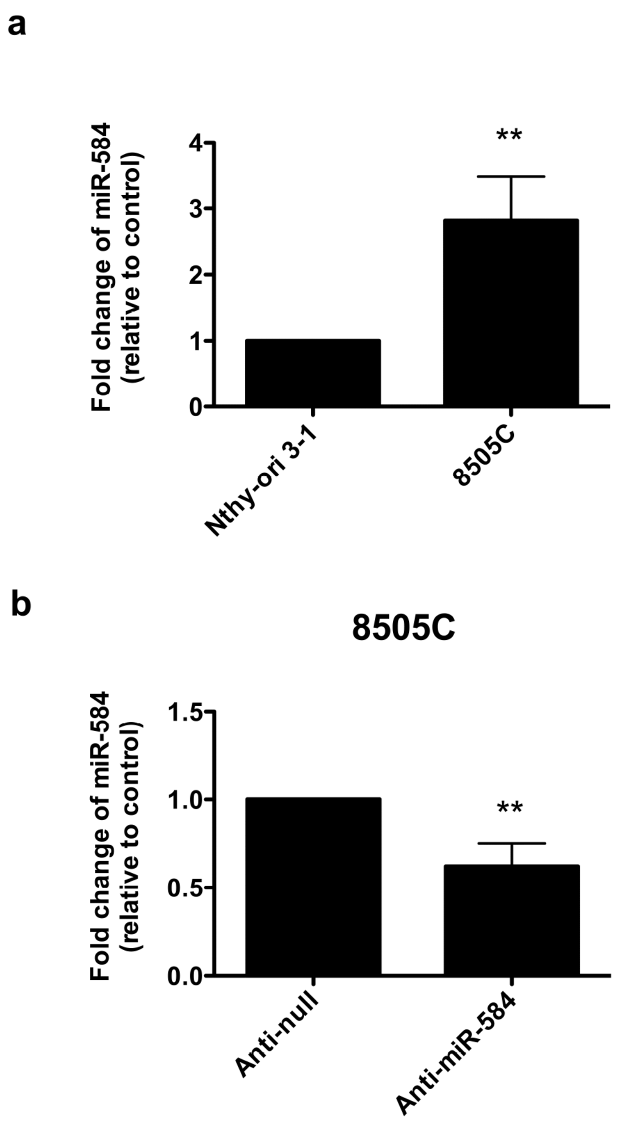

Supplementary Figure S4: Expression level of miR-584 in 8505C cell line. a. The basal level of miR-584 in 8505C cells was measured, by qRT-PCR, compared to Nthy-ori 3-1 cells. The expression levels of miR-584 were normalized to those of U6 snRNA. The average results of three independent experiments ± SD are plotted (**, $p < 0.01$). **b.** Silencing of miR-584 in 8505C cells was measured by qRT-PCR after stable transfection with anti-miR-584 or empty vector (anti-null). The expression levels of miR-584 were normalized to those of U6 snRNA. Values represent the average of three independent experiments ± SD (**, $p < 0.01$).

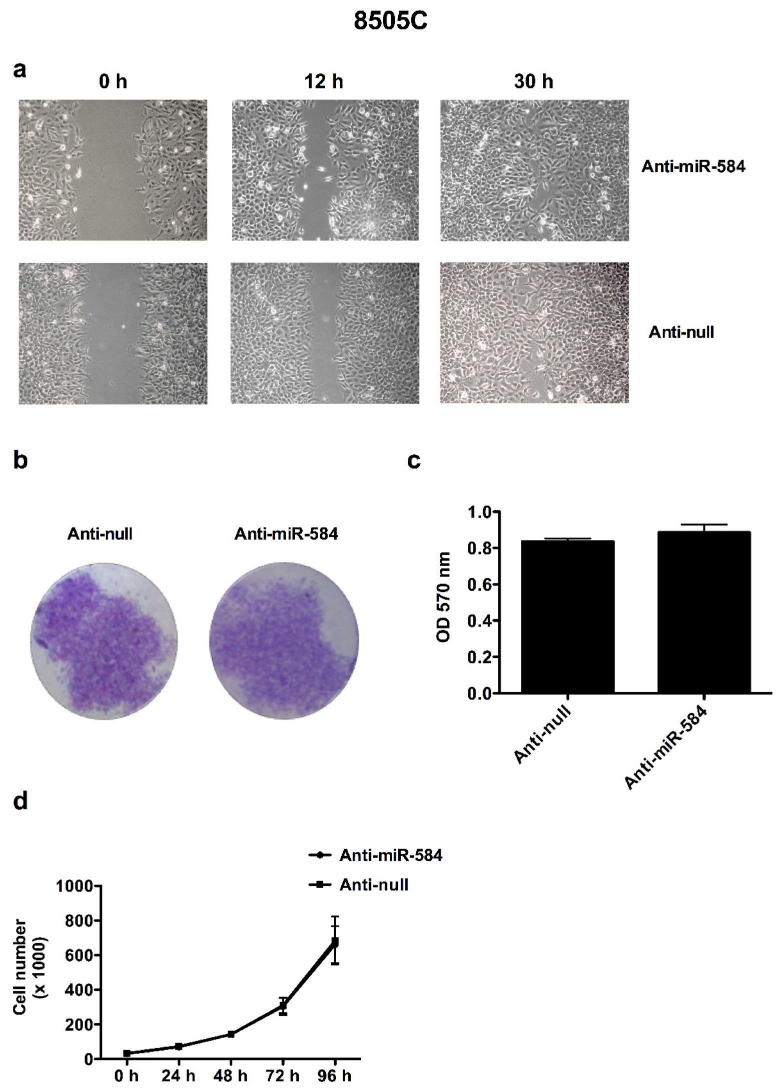

Supplementary Figure S5: Effects of miR-584 silencing in 8505C cells on cell migration, invasion and proliferation. **a.** Scratch wounds were inflicted at confluent monolayer of 8505Ccells transfected with anti-miR-584 or anti-null and after 12 and 30 hours cells were photographed. **b-c**. Cells were seeded in upper chamber of transwells, allowed to migrate for 24 hours, stained and photographed (b); cell invasion ability is expressed as absorbance at O.D. 570 nm (c). **d.** 8505Ccells, transfected with anti-miR-584 or anti-null, were plated and counted in triplicate every 24 hours for 4 days. Values represent the average of three independent experiments ± SD.

Supplementary Table S1: miRNAs up-regulated in TPC-Twist1 cells

	pcDNA	Twist1 mp1		Twist1 mp2		Twist1 Cl2	
**miRNA**	**Ct**	**Ct**	**Fold Change**	**Ct**	**Fold Change**	**Ct**	**Fold Change**
hsa-mir-597	38.4	32.1	81.4	31.9	92.9	32.0	89.3
hsa-mir-29b-2*	38.8	32.9	61.6	32.6	74.7	32.5	78.1
hsa-mir-145	35.4	30.8	24.2	32.3	8.4	31.3	17.2
hsa-mir-584	38.5	35.1	10.1	31.6	116.9	32.4	68.2
hsa-mir-30c-2*	35.9	32.6	10.0	32.3	12	29.6	78.5
hsa-mir-519b-3p	38.2	35.2	7.9	36.4	3.3	34.9	9.8
hsa-mir-101	37	34.3	6.6	34.7	4.9	34.1	7.5
hsa-mir-449a	36	33.6	5.3	35.1	2	34.7	2.6
hsa-mir-501-5p	35.2	32.8	5.2	30.9	19	32.7	5.6
hsa-mir-650	35.3	33.5	3.6	34.5	1.8	34	2.5
hsa-mir-639	35.8	34.3	2.8	33.8	3.9	33.7	4.3
hsa-mir-190b	33.6	32.1	2.7	32.4	2.2	31.5	4.4
hsa-mir-500	31.4	30	2.7	30.3	2.2	29.4	3.9
hsa-mir-99a*	32.5	31.4	2.1	29.3	9	30.6	3.6

Supplementary Table S2: miRNAs down-regulated in TPC-Twist1 cells

	pcDNA	Twist1 mp1		Twist1 mp2		Twist1 Cl2	
**miRNA**	**Ct**	**Ct**	**Fold Change**	**Ct**	**Fold Change**	**Ct**	**Fold Change**
hsa-mir-326	33.6	38	21.3	37.5	14.6	Undetermined	
hsa-mir-124	34.2	38	14.2	34.7	1.4	36.1	3.7
hsa-mir-604	35.8	39.5	12.3	Undetermined		36.2	1.8
hsa-mir-222*	34.6	38	10.5	35.6	2	38.9	19.8
hsa-mir-190	33.5	36.9	10.1	34.3	1.7	34.3	1.8
hsa-mir-770-5p	35.6	38.8	9.2	37.2	3.1	37.6	3.9
hsa-mir-137	33.1	35.9	6.7	Undetermined		33.8	1.6
hsa-mir-376c	35.7	38.5	6.6	Undetermined		Undetermined	
hsa-mir-576-3p	33.4	36.1	6.6	33.8	1.3	33.8	1.4
hsa-mir-202	32.4	34.9	5.5	33.6	2.4	34.1	3.2
hsa-mir-551b*	31.5	33.8	4.9	32.9	2.8	34.6	8.6
hsa-mir-15b*	28.9	31.2	4.9	30.1	2.3	29.9	2.1
hsa-mir-30d*	33.2	35.4	4.6	37.0	14.1	33.9	1.6
hsa-mir-148b*	32.0	33.9	3.7	34.0	3.9	32.6	1.5
hsa-mir-363	35.7	37.6	3.7	38.9	9.0	Undetermined	
hsa-mir-200c	29.4	31.3	3.6	30.4	2.0	32.8	10.1
hsa-mir-100*	30.5	32.3	3.6	31.8	2.4	33.2	6.6
hsa-mir-301a	30.8	32.7	3.6	31.4	1.5	34.9	17.3
hsa-mir-331-5p	31.8	33.6	3.5	33.7	3.8	33.2	2.6
hsa-mir-215	29.5	31.2	3.5	30.2	1.7	33.1	12.8
hsa-mir-206	31.7	33.4	3.3	32.6	1.9	31.9	1.2
hsa-mir-572	31.0	32.7	3.2	31.4	1.3	31.6	1.6
hsa-mir-622	33.3	34.8	3.0	34.3	2.0	35.9	6.4
hsa-mir-579	33.9	35.4	2.9	Undetermined		Undetermined	
hsa-mir-432*	29.1	30.6	2.7	29.8	1.6	30.5	2.6
hsa-mir-505	31.4	32.8	2.7	31.6	1.2	32.8	2.7
hsa-mir-888	37.0	38.3	2.5	37.8	1.8	38.5	2.9
hsa-mir-194	27.6	28.7	2.3	28.3	1.6	29.6	4.1
hsa-mir-340	29.6	30.7	2.1	31.0	2.5	30.7	2.1
hsa-mir-218	30.4	31.4	2.1	30.7	1.3	32.4	4.1
hsa-mir-184	31.9	32.9	2.1	33.7	3.6	36.4	22.8

Supplementary Table S3: Clinicopathological features of papillary thyroid carcinomas (PTC) samples used in immunohistochemistry analysis

PTC	Age	Sex	Tumor size (cm)	Distant metastasis	Multifocal tumor	Tumor capsule infiltration	Diffuse parenchymal	Extrathyroid extension
1	52	M	1.5	NO	YES	YES	YES	NO
2	50	F	1	NO	YES	YES	YES	NO
3	42	F	1.5	NO	YES	YES	YES	NO
4	44	F	1	NO	NO	YES	YES	YES
5	55	F	1.8	NO	NO	YES	YES	YES
6	56	F	1.3	YES	YES	YES	YES	NO
7	16	M	1.5	NO	NO	YES	YES	NO
8	58	M	1.3	NO	YES	YES	YES	NO
9	63	M	1.6	YES	YES	YES	YES	YES
10	39	F	1.1	NO	NO	YES	YES	NO
11	35	M	1	YES	NO	YES	YES	YES
12	69	M	2.5	YES	YES	YES	YES	NO
13	30	F	0.8	NO	NO	YES	YES	NO
14	25	M	1.2	YES	YES	YES	YES	NO
15	42	F	3.6	YES	YES	YES	YES	NO
16	44	M	1.3	NO	YES	YES	YES	NO
17	35	F	1.6	NO	NO	YES	YES	NO
18	26	F	4	NO	NO	NO	NO	NO
19	41	M	6.5	YES	NO	YES	YES	YES
20	51	F	0.5	NO	NO	YES	YES	YES
